# Gastric lipoma of the pylorus – case report of an incidental finding

**DOI:** 10.1016/j.radcr.2021.04.054

**Published:** 2021-05-25

**Authors:** Aleksandar Georgiev, Silvia Tsvetkova, Lyubka Aleksova, Metin Ali

**Affiliations:** a Department of Diagnostic Imaging, Medical University Plovdiv, Bul. Vasil Aprilov 15A, Plovdiv, Bulgaria; b Complex Oncology Center Plovdiv, "Pere Toshev" str., Plovdiv, Bulgaria; c Department of Special Surgery, Medical University Plovdiv, Bul. Vasil Aprilov 15A, Plovdiv, Bulgaria

**Keywords:** Gastric Lipoma, Pyloric Lipoma, Benign stomach neoplasms, CT

## Abstract

Gastric lipomas are very rare benign tumors. Only around 217 cases have been reported. Most gastric lipomas are found incidentally; however, larger neoplasms can be symptomatic. The presented 64 years old male with incidental finding of pyloric lipoma adds another example to the few documented in the literature. The patient had symptoms of breath shortness and lack of energy two months after COVID 19 pneumonia. A low dose CT scan with iodine contrast enhancement of the chest and upper abdomen led to the diagnose. The diagnosis of gastric lipoma can be achieved through diagnostic imaging or the combination of endoscopic techniques. Treatment can be carried out by endoscopy, robotic or classical surgery.

## Introduction

Gastric lipomas are rare tumors, comprising only 1-3% of benign stomach tumors [1]. Most gastric lipomas are found incidentally; however, larger tumors can be symptomatic. Upper gastrointestinal dyspeptic symptoms and bleeding are the most commonly reported clinical signs [2,3]. CT findings of gastric lipomas are identical to ordinary extra gastric lipomas. Typically, they are well-demarcated, oval lesions with low density- composed of fat tissue. Gastric lipomas are solitary in 75% of the described cases and are most frequently located in the gastric antrum. They tend to be submucosal rather than subserosal [4], [5], [6]. Management of symptomatic lipomas is traditionally endoscopic excision in small lesions with larger lesions undergoing classical surgery, although this is debated [5], [6], [7]. The presented incidental finding of pyloric lipoma adds another example to the few documented in the literature.

## Case report

The reported case is of a 64-year-old man. The patient had symptoms of breath shortness and lack of energy two months after COVID 19 pneumonia. 256 Helical CT scan with low dose protocol and iodine contrast enhancement of the chest and upper abdomen is performed. After the scan and the incidental finding of the pyloric lipoma, the patient was questioned about clinical symptoms. He described several instances of heartburn and epigastric pain after meals in the last year. He has not experienced vomiting or other upper dyspeptic symptoms.

**Fig. 1:**
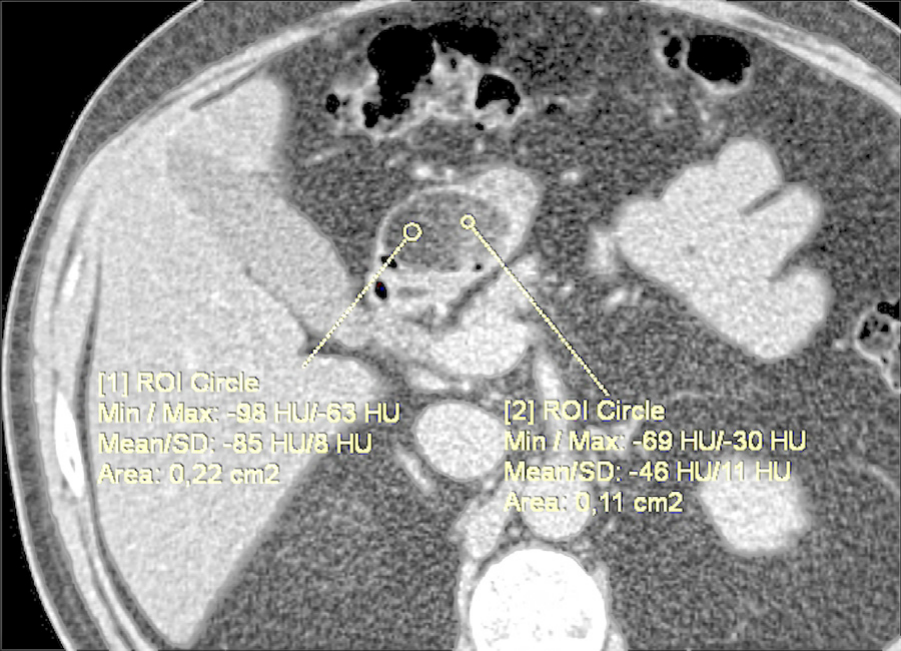
In an axial view, in the area of the pylorus of the stomach, an oval-shaped formation with a fatty density characteristic, sharp and smooth outlines is visualized.

**Fig. 2:**
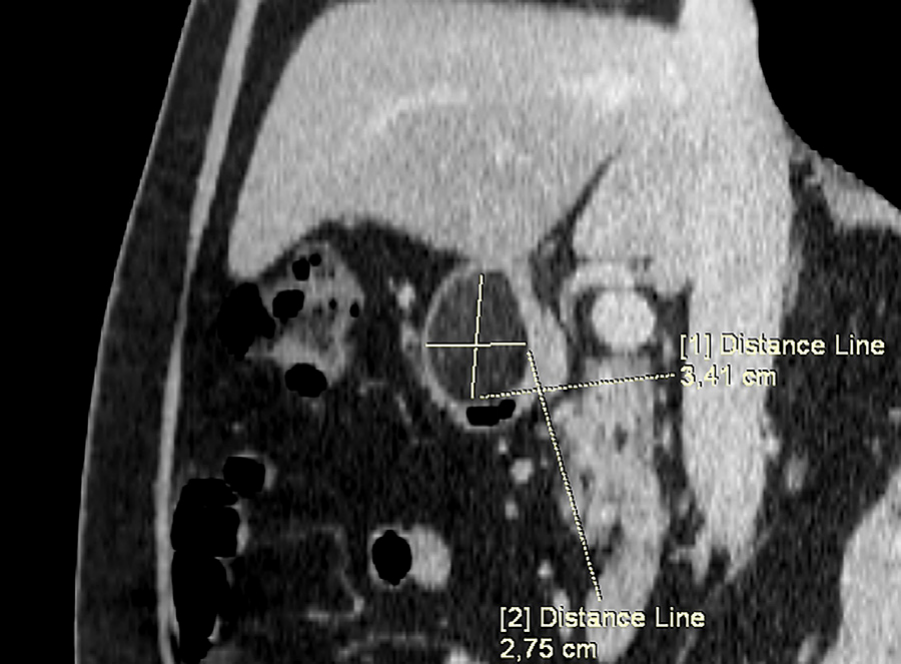
In coronary view, the  known formation in the pylorus with measurements of cranio-caudal and ventro-dorsal size.

**Fig. 3:**
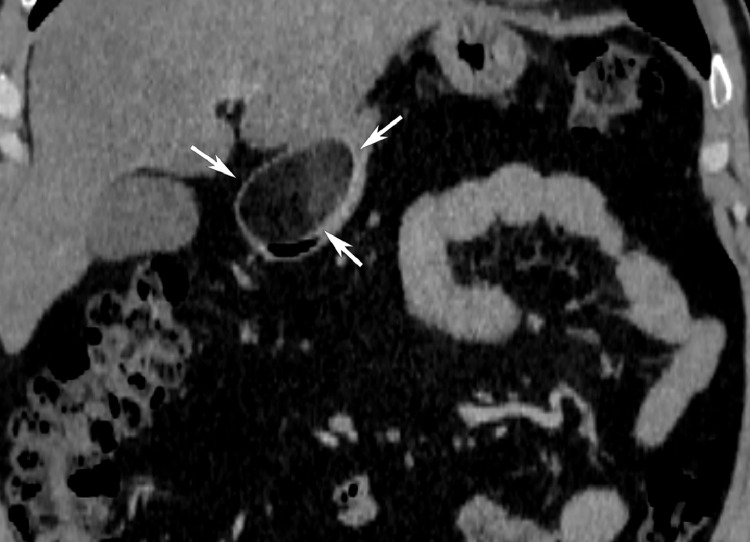
In oblique view, the described pyloric formation (white arrows) is at the level of the gallbladder and the right kidney - there is no evidence of ptosis and dilatation of the stomach.

The CT scan of the upper abdomen shows an oval, well-encapsulated formation in the pylorus of the stomach. Due to the contrast enhancement, the lesion appears non homogeneously contrasted but remains with fatty density. Measurements of CT density after contrast enhancement in Hounsfield Units (HU) are between -85 and -46 in different locations of the lipoma. (Fig. 1). The formation is not small 3.41/2.75 cm. but does not entirely obstruct the pyloric channel. (Fig. 2) The stomach does not appear to be dilatated with no visible sign of chronic pyloric stenosis. (Fig 3) The chest CT scan reveals no signs of pathology. No "ground-glass" opacities or fibrotic changes are detected in the lungs. No pleural or pericardial effusion is visible on the chest scans and no visible sign of pulmonary embolism.

After the incidental finding and CT diagnosis of pyloric lipoma, the patient is advised to consult an abdominal surgeon for histological verification and treatment planning.

## Discussion

Gastric lipomas are very rare, slow-growing benign tumors formed by mature adipose tissue. The colon is the most common site of involvement within the gastrointestinal tract, followed by the ileum and jejunum [6,^8^,9]. Only around 217 cases of gastric lipomas have been reported in the literature.

Lipomas close to the pylorus, such as in the presented case, can cause obstructive symptoms. They can be caused by blocking the pylorus or protruding into the duodenum. It is reported that a lipoma of a size greater than 2.0 cm will present with abdominal pain more than 50% of the time; however, 37% of patients will have a presentation of chronic or acute GI bleeding, obstruction, and dyspepsia [2,^7^,^8^,10]. But as diseases rarely read the literature, the reported neoplasm has a larger size and very mild clinical manifestation.

The most important differential diagnosis of gastric lipoma is liposarcoma. Differential diagnosis can also be made with soft tissue tumors of the gastrointestinal tract, such as leiomyoma or fibroma, neurilemoma adenomyoma, Brunner gland adenoma, and ectopic pancreas [10], [11], [12]. Histologically, there are four types of liposarcomas: well-differentiated, myxoid, round cell, and pleomorphic. Well-differentiated liposarcomas account for 40% of all liposarcomas. Their peak incidence is between the 5th and 7th decades [2,^7^,^13^,14].

Usually, on barium studies, extra mucosal neoplasms, including lipomas, appear as a well-defined filling defect with a 'bull's eye' appearance.3 CT is a precise imaging diagnostic tool for lipoma [8,15]. The CT is beneficial for its diagnosis. The neoplasm appears as a well-defined tumor, of a density, with attenuation ranging from−70to−120HU on native scans, which is pathognomonic in these cases [2,^3^,^8^,9]. The choice of treatment for gastric lipomas is still controversial. Various surgical and endoscopic procedures have been used in the treatment of submucosal lipomas.

Upper GI endoscopy will show a submucosal mass and three signs which help diagnosis [10,^11^,16]. These are the tenting sign, the cushion sign, and the naked fat sign and are characteristics of gastric lipomas. The tenting sign occurs when the overlying mucosa can be easily retracted with the biopsy forceps. The cushion sign is demonstrated when the forceps leave a mark when pressed against the lipoma. The naked fat sign is the visible, exposed fatty tissue on the surface of the lipoma [12,13]. Endoscopic biopsy is usually inconclusive since the tumor is frequently submucosal.

## Conclusions

In some cases, CT may produce a diagnosis without having to perform an endoscopy. More accurate diagnosis of gastric lipoma can be achieved through the combination of diagnostic imaging and endoscopic techniques. Treatment can be carried out by endoscopy, robotic or classical surgery, depending on the specific case.

## Patient consent statement

Patient consent has been obtained.
